# Unlocking the genomic potential of Red Sea coral probiotics

**DOI:** 10.1038/s41598-024-65152-8

**Published:** 2024-06-24

**Authors:** Inês Raimundo, Phillipe M. Rosado, Adam R. Barno, Chakkiath P. Antony, Raquel S. Peixoto

**Affiliations:** Biological and Environmental Science and Biological and Environmental Science and Engineering Division (BESE), King Abdullah University of Science and Technology, Biological and Environmental Science and Engineering Division, Thuwal, Saudi Arabia

**Keywords:** Coral, Probiotics, Genome, Prophage, Secondary metabolites, Beneficial microorganisms for corals, Pacbio, Microbial ecology, Water microbiology

## Abstract

The application of beneficial microorganisms for corals (BMC) decreases the bleaching susceptibility and mortality rate of corals. BMC selection is typically performed via molecular and biochemical assays, followed by genomic screening for BMC traits. Herein, we present a comprehensive in silico framework to explore a set of six putative BMC strains. We extracted high-quality DNA from coral samples collected from the Red Sea and performed PacBio sequencing. We identified BMC traits and mechanisms associated with each strain as well as proposed new traits and mechanisms, such as chemotaxis and the presence of phages and bioactive secondary metabolites. The presence of prophages in two of the six studied BMC strains suggests their possible distribution within beneficial bacteria. We also detected various secondary metabolites, such as terpenes, ectoines, lanthipeptides, and lasso peptides. These metabolites possess antimicrobial, antifungal, antiviral, anti-inflammatory, and antioxidant activities and play key roles in coral health by reducing the effects of heat stress, high salinity, reactive oxygen species, and radiation. Corals are currently facing unprecedented challenges, and our revised framework can help select more efficient BMC for use in studies on coral microbiome rehabilitation, coral resilience, and coral restoration.

## Introduction

Coral reefs are one of the most diverse and productive ecosystems on Earth. However, they are currently facing unprecedented challenges due to overfishing, pollution, and climate change^1–4^. Increased greenhouse gas concentrations leading to global warming^5^, pollution and ocean acidification have emerged as key stressors, threatening the survival of coral reefs worldwide^3,6–8^. Some of these stressors cause periods of high seawater temperatures, leading to the loss of Symbiodiniaceae, the algal symbionts that reside within the holobiont and fulfill up to 90% of the corals’ nutritional requirements^9^ except when they become nutrient competitors and can be expelled^10–12^. This loss, especially over a long-term, can lead to devastating bleaching events worldwide, in which corals are deprived of vital nutrients, leading to increased disease susceptibility and reduced reproductive success and skeletal growth^2,13–16^. This disruption extends beyond the loss of algal symbionts because it is followed by shifts in the composition and function of the entire coral-associated microbiome^17^. This causes post-heat stress disorder^18^ that compromises the coral’s ability to grow, resist pathogens, modulate nutrient cycling, and maintain holobiont homeostasis^18–27^.

The symbiotic relationship between corals and their bacterial symbionts^28,29^ plays a pivotal role in maintaining the health of these organisms and consequently the functioning of reef ecosystems. Although some corals and their microbiomes have shown the capacity to recover from acute stress events^30,31^, the coral microbiome may not fully restore its original composition and functionality following a severe disturbance^32^.

Rehabilitation of the coral microbiome^33^ has recently emerged as a promising strategy to enhance coral health and resilience^18,24, 26, 33–42^. Coral-associated bacteria possess potential beneficial traits that enhance the fitness and resilience of their coral host by maintaining homeostasis through their involvement in various essential processes, such as nutrient cycling, production of antibiotics and antimicrobial compounds, and mitigation of toxic compounds^33,43^. Harnessing the symbiotic interactions between corals and their associated bacteria^28^ allows researchers to selectively introduce these beneficial microorganisms for corals (BMC)^33^ into the coral holobiont for use as customized probiotics. This can help maintain or even enrich the proportion of native beneficial microorganisms to enhance coral health and resilience under stress^33,35^.

In this study, we performed an in silico investigation of the probiotic potential of six putative BMC (pBMC) strains isolated from corals in the Red Sea with the aim of developing the first Red Sea BMC consortium. This was achieved by analyzing the complete genomes of all six strains and screening for the presence of potential beneficial mechanisms for corals. Our research provides a framework for the selection of novel, customized BMC consortia based on the presence of particular BMC characteristics that support host health and survival under stress conditions.

## Materials and methods

### pBMC isolation and genome sequencing

pBMC strains were isolated from coral fragments (± 5 cm long) of *Pocillopora verrucosa, Galaxea fascicularis,* and *Stylophora pistillata* Clade IV. The fragments were collected between February and April 2021 from Al Fahal reef (22°18′18.4′′N, 38°57′52.5′′E), Aquarium reef (22°23′15.6′′N, 38°55′07.2′′E), and Tahala reef (22°15′46.9′′N, 39°03′05.9′′E) in the eastern central Red Sea, Saudi Arabia, as described by Delgadillo-Ordoñez and colleagues^44^.

Briefly, coral fragments were macerated in 50 mL of 3.5% sterile saline solution using a sterile mortar and pestle. Then, the macerated paste was serially diluted up to 10^−6^ with 3.5% saline solution, and 100 µL of each dilution was plated on Zobel Marine Agar 2216 (MA; HiMedia Laboratories, Mumbai, India), diluted marine agar (DMA; 2 × dilution of MA with 3.5% NaCl and adjusted agar content) and Lennox Broth (Sigma-Aldrich) and cultured overnight at 25 °C. The macerate was also incubated in parallel at 25 °C overnight and 130 rpm in a 250-mL sterile Erlenmeyer flask containing crystal beads. After this first incubation, 100 µL triplicate subsamples of 10^−4^, 10^−5^, and 10^−6^ dilutions were plated on approximately 20 mL of MA and DMA. Alternatively, 0.5-cm long coral fragments were plated on both media. All plates were incubated at 25 °C for at least 48 h or until visible bacterial colonies were observed. Based on colony morphology, > 350 isolates were obtained, which were preserved at −80 °C in sterilized 20% glycerol. To select pBMC strains, the isolates were screened for various beneficial traits using polymerase chain reaction (PCR) assays targeting nitrogen cycle-related genes involved in nitrogen fixation (*nifH*) and denitrification (*nirK*) as well as biochemical analyses of catalase activity, siderophore production^45^ and phosphate assimilation^46^. Finally, the antagonistic effect of the isolates against the coral pathogen *Vibrio coralliilyticus*^47,48^ was tested following the methodology described by Rosado and colleagues^26^ and Santoro and colleagues^18^. Based on the positive results of these tests, six strains were selected as pBMC and stored in glycerol stocks (20%) at −80 °C.

We extracted genomic DNA (gDNA) from these six pBMC strains using Wizard Genomic DNA Purification Kit (Promega, Madison, WI, USA) according to the manufacturer’s protocol. The resultant gDNA was purified with AMPure beads (1:8 ratio; Beckman Coulter) and quantified using Qubit dsDNA Assay Kit (Invitrogen). Then, the quality and integrity of the extracted gDNA were confirmed via spectrometry using a NanoDrop 8000 spectrophotometer (Thermo Fisher Scientific) and via electrophoresis using the 4200 TapeStation System (Agilent Technologies). Next, we prepared SMRTbell libraries using SMRTbell Express Template Prep Kit 2.0 (Pacific Biosciences) and Barcoded Overhang Adapter Kit 8A (Pacific Biosciences) according to the PacBio multiplexed microbial libraries protocol. The quality of the libraries was assessed using Qubit dsDNA High Sensitivity Assay Kit (Thermo Fisher Scientific) and FEMTO Pulse pulsed-field capillary electrophoresis system (Agilent Technologies). For sequencing, the libraries were first annealed using Primer v4 via Sequel Binding Kit 3.0 (Pacific Biosciences) and then sequenced using SMRT Cell 1 M v3 LR Tray (Pacific Biosciences) via the Sequel system at the Bioscience Core Lab of King Abdullah University of Science and Technology, Saudi Arabia.

### pBMC genome assembly and taxonomic classification

The quality of raw reads was assessed using LongQC tool^49^, and the reads were trimmed according to the default parameters. Next, the reads were assembled using Canu^50^, Flye^51^, Raven^52^, Miniasm/Minipolish^53^, and Redbean^54^. The generated assemblies were then evaluated and compared to determine the quality of general genome assembly via Quast^55^, genomic completeness/contamination via CheckM software^56^, chimerism via GUNC^57^, and indel errors/pseudogenes via ideel (https://github.com/phiweger/ideel). The assemblies from Canu were considered the best based on assembly quality, genomic completeness, and lower number of indels/interrupted open reading frames. FastANI^58^, and Genome-to-Genome Distance Calculator^59^ were used to assess the phylogenetic relatedness between the study genomes and the publicly available closest reference strain genomes.

### Structural and functional annotation of pBMC genomes

The six pBMCs genomes were annotated using Prokka^60^ and the PATRIC Bacterial and Viral Bioinformatics Resource Center (BV-BRC)^61^, which uses the RASTtk online server^62^, according to the default parameters. The output data were visualized on the BV-BRC interface. We screened for protein-coding genes that might provide beneficial characteristics for host health and resilience^33,43^ by searching the subsystems generated by the RASTtk platform in BV-BRC. Additionally, the six pBMC genomes were searched for putative prophage sequences using the PHASTEST and VirSorter2 online analysis tools^63,64^. PHASTEST analyzes the proportion of phage proteins within a bacterial genomic region and scores the potential phages based on their length and inclusion of several phage-related proteins, such as “capsid,” “head,” “integrase,” “plate,” “tail,” “fiber,” “coat,” “transposase,” “portal,” “terminase,” “protease,” or “lysin,” in the input file. VirSorter2 uses a set of viral HMM databases created from various large-scale viral metagenome projects and trained viral classifiers to identify viral sequences within predominantly metagenomic samples. Prophages detected by both tools were further analyzed using CheckV^65^. Only the consensus prophages characterized as high quality by both identification tools were used in this analysis. Only phages considered intact based on the abovementioned criteria were used for further analysis. Intact phages were queried using the IMG/VR v4 online BLAST database, and similarity was assessed based on the highest reported bit score with high-quality reference phages^66^. AntiSMASH v7^67^ was also used to identify, annotate, and analyze secondary metabolite biosynthetic gene clusters (BGCs) present in the genomes of all six pBMC strains.

### Phylogenomic trees

**Figure 1:**
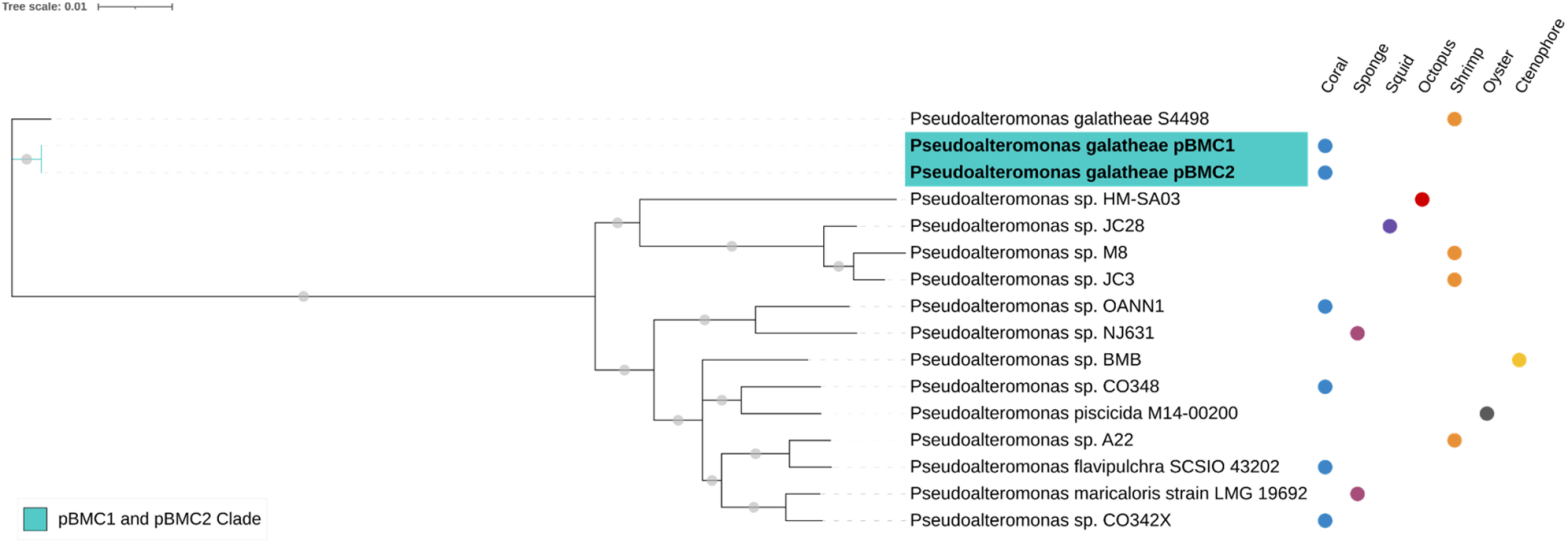
Phylogenomic inference of publicly available genomes from 14 *Pseudoalteromonas* sp. strains and the genomes of our two *Pseudoalteromonas galatheae* strains (pBMC1 and pBMC2; highlighted in bold), totaling 16 genomes. The tree was assembled by comparing 1000 proteins using the codon tree method of the PATRIC platform. This method selects global protein families as homology groups and analyzes aligned proteins and DNA encoding single-copy genes using the RAxML program. Based on RAxML, the Jones–Taylor–Thornton protein model was considered the best model to construct this tree. Gray dots on the branches correspond to a bootstrap value of 100. The isolate source is indicated on the right side of each strain. The blue-shaded area represents the monophyletic clade comprising pBMC1 and pBMC2.

**Figure 2:**
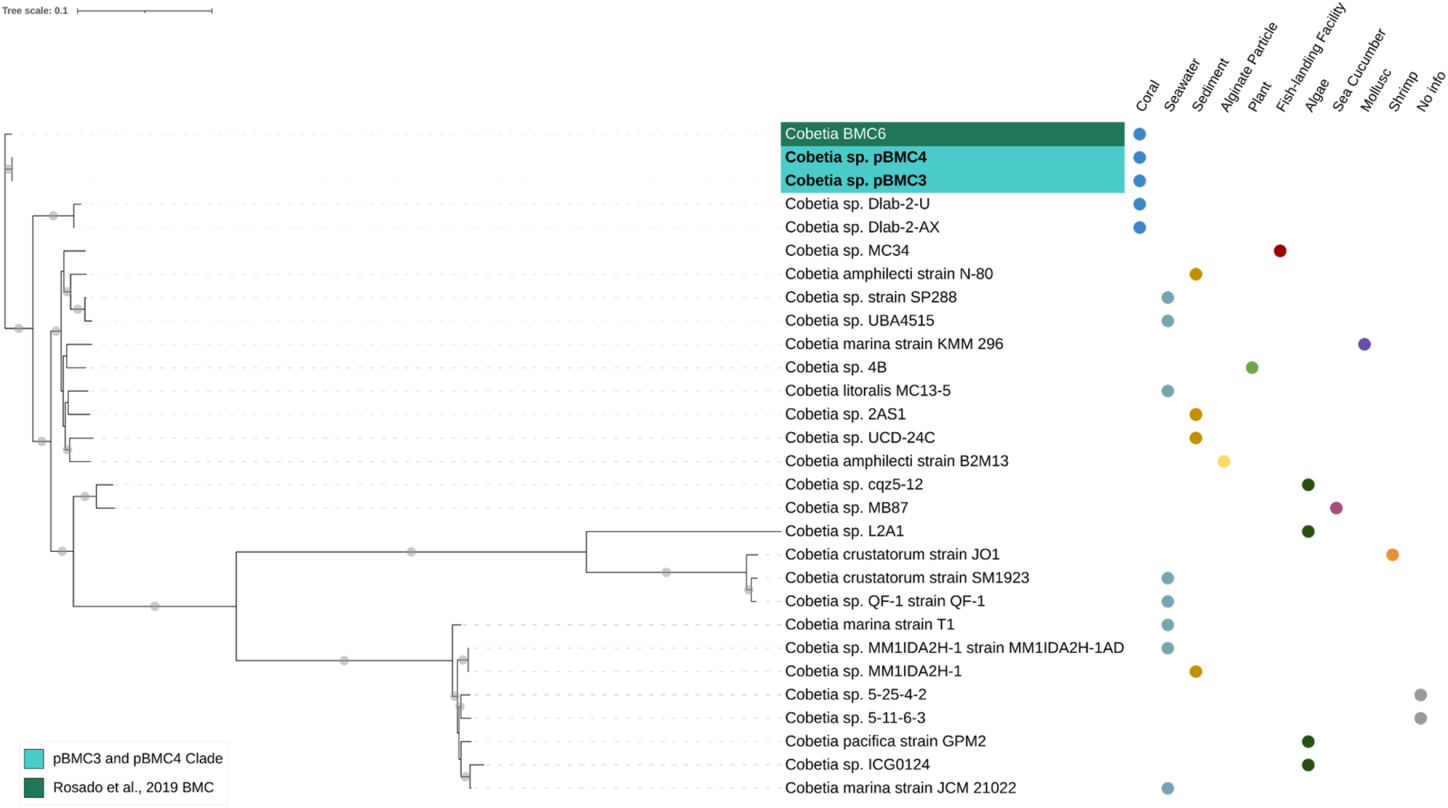
Phylogenomic inference of publicly available genomes from 26 *Cobetia* sp*.* strains, the genome of *Cobetia* sp. BMC6 from Rosado and colleagues^172^, and the genome of our two *Cobetia* sp. strains (pBMC3 and pBMC4; highlighted in bold) totaling 29 genomes. The tree was assembled by comparing 1000 proteins using the codon tree method of the PATRIC platform. This method selects global protein families as homology groups and analyzes aligned proteins and DNA encoding single-copy genes using the RAxML program. Based on RAxML, the Jones–Taylor–Thornton protein model was considered the best model to construct this tree. The isolate source is indicated on the right side of each strain. Gray dots on the branches correspond to a bootstrap value of 100. The blue-shaded area represents the monophyletic clade comprising pBMC3 and pBMC4. The *Cobetia* sp. BMC strain from the study by Rosado and colleagues^172^ is shaded in green.

**Figure 3:**
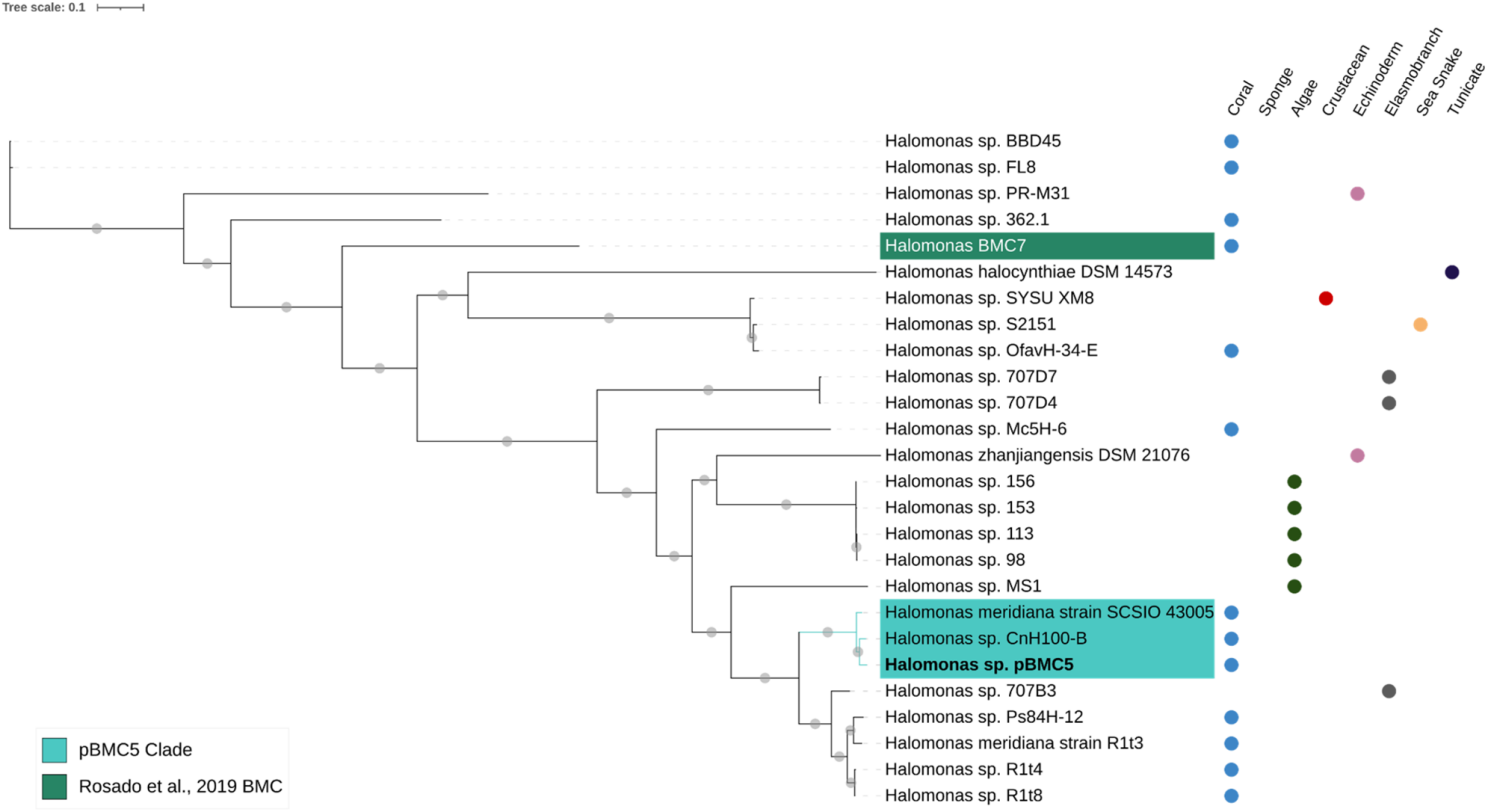
Phylogenomic inference of publicly available genomes from 24 *Halomonas* sp. strains, the genome of *Halomonas* sp. BMC7 from Rosado and colleagues^172^ and the genome of our *Halomonas* sp. strain pBMC5 (highlighted in bold), totaling 26 genomes. The tree was assembled by comparing 1000 proteins using the codon tree method of the PATRIC platform. This method selects global protein families as homology groups and analyzes aligned proteins and DNA encoding single-copy genes using the RAxML program. Based on RAxML, the LG model was considered the best model to construct this tree. Gray dots on the branches correspond to a bootstrap value of 100. The isolate source is indicated on the right side of each strain. The blue-shaded area represents the strains that form a monophyletic clade with pBMC5. The *Halomonas* sp. BMC strain from Rosado and colleagues^26,172^ is shaded in green.

**Figure 4:**
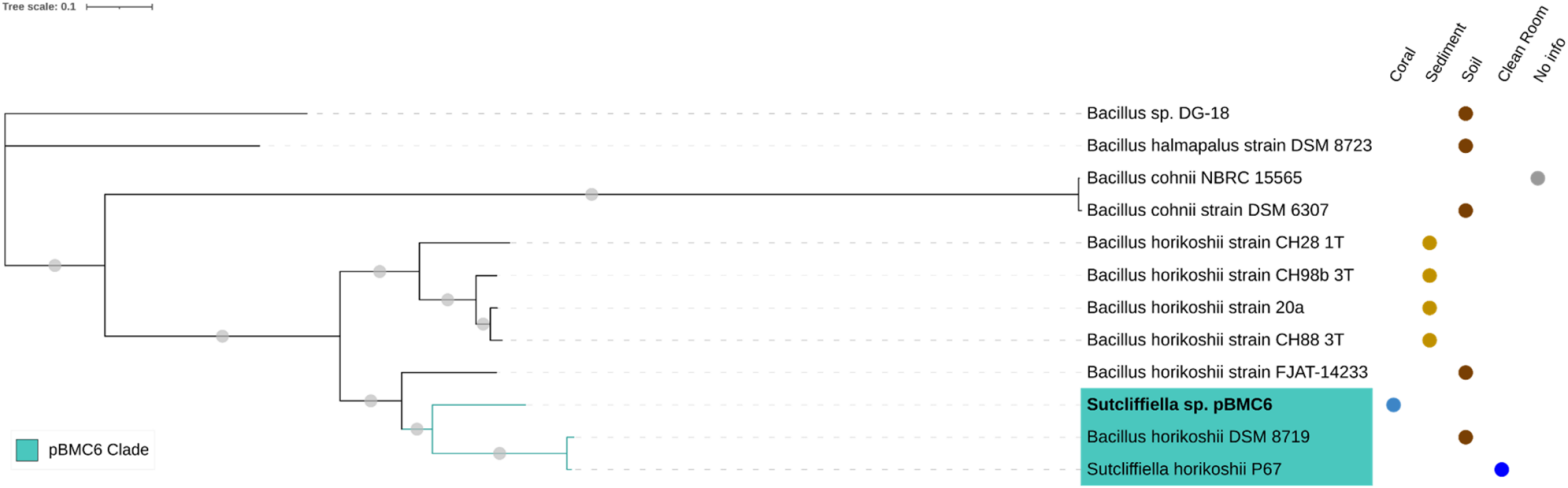
Phylogenomic inference of publicly available genomes from 1 *Sutcliffiella* sp. strain and ten *Bacillus* sp. strains and the genome of our *Sutcliffiella* sp. strain pBMC6 (highlighted in bold), totaling 12 genomes. The tree was assembled by comparing 1000 proteins using the codon tree method of the PATRIC platform. This method selects global protein families as homology groups and analyzes aligned proteins and DNA encoding single-copy genes using the RAxML program. Based on RAxML, the LG model was considered the best model to construct this tree. Gray dots on the branches correspond to a bootstrap value of 100. The isolate source is indicated on the right side of each strain. The blue-shaded area represents the strains that form a monophyletic clade with pBMC6.

**Figure 5:**
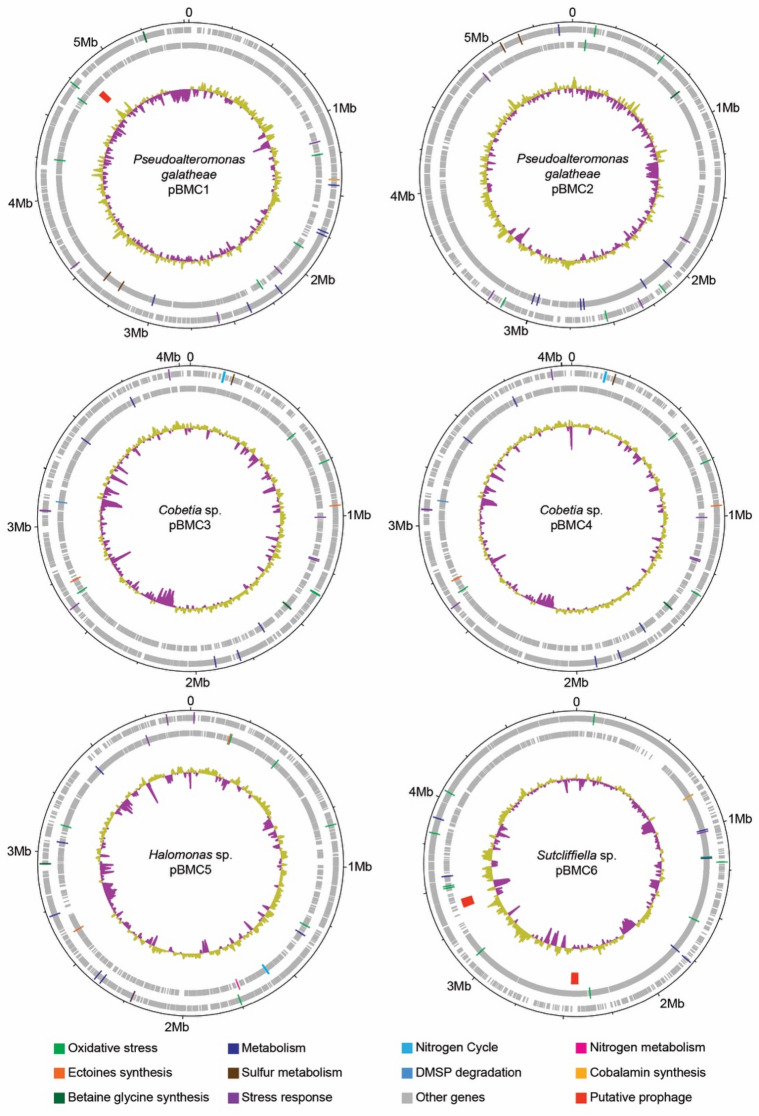
Genome maps of the six putative BMC strains. The rings (from outside to inside) indicate the nucleotide position in the genome, predicted genes on the forward strand, predicted genes on the reverse strand, location of putative prophages (as predicted by PHASTEST and VirSorter2), and percent GC content (higher GC content = yellow, lower GC content = purple). Genes predicted to encode beneficial proteins are color-coded in the outer rings. The circular genomes were visualized using DNAplotter ^178^.

A phylogenomic assemblage was constructed using the codon tree method via the BV-BRC platform. This method randomly selects up to 10 proteins to represent each genus-level family and merges them to create a single set of representatives, preventing cluster formation, which is based on genus similarity rather than protein similarity (PATRIC global protein families [PGFams])^61,68^. Amino acids and nucleotide sequences were used for analyzing each selected gene from the PGFams. Protein sequences were aligned using MUSCLE^69^, and encoding gene sequences were aligned using the Codon_align function in BioPython^70^. The output was a concatenated alignment of all proteins and nucleotides as well as a partitioned file describing the alignment of proteins for analysis using RAxML^71^. Support values were generated using 100 rounds of the “Rapid” bootstrap option^72^ in RAxML. The phylogenomic trees were constructed by comparing and analyzing protein-coding genes (*n* = 1000, Figs. 1, 2, 3, 4 and 5; *n* = 500, Fig. S1) shared among all genomes using the best protein model method identified by RAxML^73^. The output, in Newick format, was then visualized via iTOL version 6^74,75^. The trees generated are separated by genus (*Pseudoalteromonas*, *Cobetia*, *Halomonas*, and *Sutcliffiella*), and the genomes used to construct these trees were selected from the BV-BRC platform database based on various criteria, such as being reference genomes, complete genomes, and isolated from corals and/or aquatic environments, as per their availability.

### Pangenomic analysis and gene categorization

We used the Prokka annotations to generate a separate pangenome for each pBMC genus (*Pseudoalteromonas, Cobetia, Halomonas,* and *Sutcliffiella*) using the Roary tool^75^, with the default settings. For each pangenome, sequences from the closest genomes to our pBMC strains, which were identified based on the phylogenomic tree results, were added to the analysis. For the pangenomic analysis of *Pseudoalteromonas* sp., in addition to our two *Pseudoalteromonas galatheae* genomes (pBMC1 and pBMC2), 19 additional *Pseudoalteromonas* sp. genomes were included (from Fig. 1). For the pangenomic analysis of *Cobetia* sp., in addition to our two *Cobetia amphilecti* genomes (pBMC3 and pBMC4), three *Cobetia* sp. genomes were added (from Fig. 2). The pangenome of *Halomonas* sp. included our single *Halomonas* sp. genome (pBMC5) and three other *Halomonas* sp. genomes (from Fig. 3). For *Sutcliffiella* sp., its pangenome consisted of the genome of *Sutcliffiella* sp. pBMC6, one other *Sutcliffiella* sp. genome, and two *Bacillus* sp. genomes (from Fig. 4). All genomes used for comparison with the pBMC genomes were obtained from the BV-BRC genome database^61^. The pangenomes were created and visualized using the Anvi’o platform^76^, and the genes were categorized according to these results. Singleton genes were defined as genes that were only present in one or more of the pBMC genomes and not in the other genomes that were part of the same pangenome. These singleton genes were identified for each pBMC and screened for their potential to enhance the health status and resilience of the host using UniProt (https://www.uniprot.org/)^77^.

## Results

### Genomic features of pBMC

The six pBMC genomes were sequenced using PacBio, resulting in several cleaned and trimmed high-quality reads of 137,126–345,950, with an average read length of 5827 bp. The genomes were assembled using Canu^50^, resulting in 2–9 contigs per strain. CheckM analysis revealed a high level of genome completeness (> 99% for all genomes except *Sutcliffiella* sp. pBMC6, which was > 97.5%) and a low level of contamination (< 2.5%) for all genomes (Table S1).

The genomes of *P. galatheae* pBMC1 and pBMC2 had very similar characteristics, with a GC content of 42.94% and 42.98% and a total length of 5,528,212 and 5,474,806 bp, respectively. The genomes of *Cobetia* sp. pBMC3 and pBMC4 also had very similar characteristics, with a GC content of 62.32% and 62.31% and a total length of 4,046,628 and 4,041,826 bp, respectively (Table S1).

### Taxonomic attribution

pBMC1 and pBMC2 represented the same species, sharing an average nucleotide identity (ANI) of > 99.5% at both genome and 16S rRNA gene levels. The DNA–DNA hybridization (DDH) and ANI values with the strain *Pseudoalteromonas galatheae* were 92% and 99.17% for pBMC1 and 92.4% and 99.19% for pBMC2, respectively, suggesting that they belonged to this species (Table S2). Notably, the species-level thresholds are 70% for DDH^78^ and 95–96% for ANI^79^. Similarly, pBMC3 and pBMC4 showed an ANI of > 99.5% at the genome and 16S rRNA gene levels. Their DDH and ANI values with other *Cobetia* sp. were also high, and pBMC3 and pBMC4 were classified as *Cobetia* sp. in this study (Table S2). pBMC5 showed 97.94% ANI with the genomes of two strains of *Halomonas meridiana* (Table S2); however, compared with the type strain of *H. meridiana* (*H. meridiana* DSM5425), pBMC5 showed an ANI of < 90% and DDH of < 70%, suggesting that it is a novel species. pBMC6 also potentially represents a novel species, with the closest species genome being that of *Bacillus horikoshii* at an ANI of 85.96% (Table S2). The systematic classification of the family Bacillaceae has undergone several modifications due to the implementation of new taxonomic polyphasic techniques^80,81^, which have led to the creation of new genera from the previously classified genus *Bacillus*, including the new genus *Sutcliffiella*^81^. Considering this, the overall taxonomic classification of the six pBMC strains investigated in our study should be as follows: *P. galatheae* (*n* = 2), *Cobetia* sp. (*n* = 2), *Halomonas* sp. (*n* = 1), and *Sutcliffiella* sp. (*n* = 1).

### Phylogenomic trees

The phylogenomic relationships between the six pBMC genomes and the closest publicly available genomes were evaluated. We used 115 genomes to assemble the phylogenomic tree of *P. galatheae* pBMC1 and pBMC2 (Fig. S1). A well-supported clade, formed by pBMC1, pBMC2, and 14 other *Pseudoalteromonas* sp. strains, was established in this phylogenomic tree. Consequently, another phylogenomic tree was assembled using the genomes of this clade (Fig. 1). All 16 strains included in this new tree were host-associated. For the phylogenomic tree of *Cobetia* sp. pBMC3 and pBMC4, the genomes of 27 other *Cobetia* sp. were added, and pBMC3 and pBMC4 formed a clade (Fig. 2). The phylogenomic tree of *Halomonas* sp. pBMC5 included 25 other *Halomonas* sp. genomes (Fig. 3). The *Halomonas* sp. pBMC5 formed a small clade with one *Halomonas* sp. strain and one *H. meridiana* strain, creating a coral-associated clade. In the phylogenomic tree of *Sutcliffiella* sp. pBMC6, all 11 publicly available genomes were included (Fig. 4). There was a small clade between *Sutcliffiella* sp. pBMC6 and two other *Sutcliffiella* sp. strains (*Sutcliffiella horikoshii* and *B. horikoshii*), one of which was isolated from soil and the other from a clean room.

### Potentially beneficial gene functions

We used the BV-BRC platform to screen for genes encoding proteins that are potentially beneficial for corals^33,43, 82^ and their endosymbiotic algae^83^. Subsystems that are known to play a role in the health and resilience of corals, as described by Peixoto and colleagues^33,43^, and Matthews and colleagues^83^, were selected, and genes associated with oxidative stress, nitrogen cycle, ectoine and B-complex vitamin biosynthesis, iron and sulfur metabolism, and dimethylsulfoniopropionate (DMSP) degradation were identified (Table S3).

Within the subsystems of interest, all pBMC harbored genes related to protection from reactive oxygen species (ROS) and biosynthesis of vitamins B2, B7, and B9. pBMC1, pBMC2, pBMC5, and pBMC6 shared genes related to catalase and peroxidase activities and vitamin B12 biosynthesis. All pBMC, except for pBMC6, harbored genes involved in vitamin B6 biosynthesis. pBMC6 was the only pBMC harboring genes involved in iron acquisition. Genes related to ectoine biosynthesis were only detected in pBMC3, pBMC4, and pBMC5, whereas genes related to the nitrogen cycle were found in pBMC3, pBMC4, and pBMC5. DMSP degradation-related genes were only detected in pBMC3 and pBMC4, whereas genes related to sulfur metabolism were only identified in pBMC1 and pBMC2 (Table S3).

### Putative prophages

Of the six described pBMC strains herein, two contained prophages that were determined to be high quality by both PHASTEST and VirSorter2. Every high-quality prophage also had a 100% completeness score. One prophage was predicted in the genome of *P. galatheae* pBMC1 (Fig. 5). This prophage was most similar to the tailed phage that infects *P. piscicida* DE2-A. No putative prophages were detected in *P. galatheae* strain pBMC2, either of the *Cobetia* sp. strains (pBMC3 and pBMC4), nor *Halomonas* sp. pBMC5. However, two prophages were found in *Sutcliffiella* sp. pBMC6, and they were most similar to the tailed phages infecting *S. horikoshii* FJAT-14233 and *S. horikoshii* DSM 8719.

### Pangenomes

**Figure 6:**
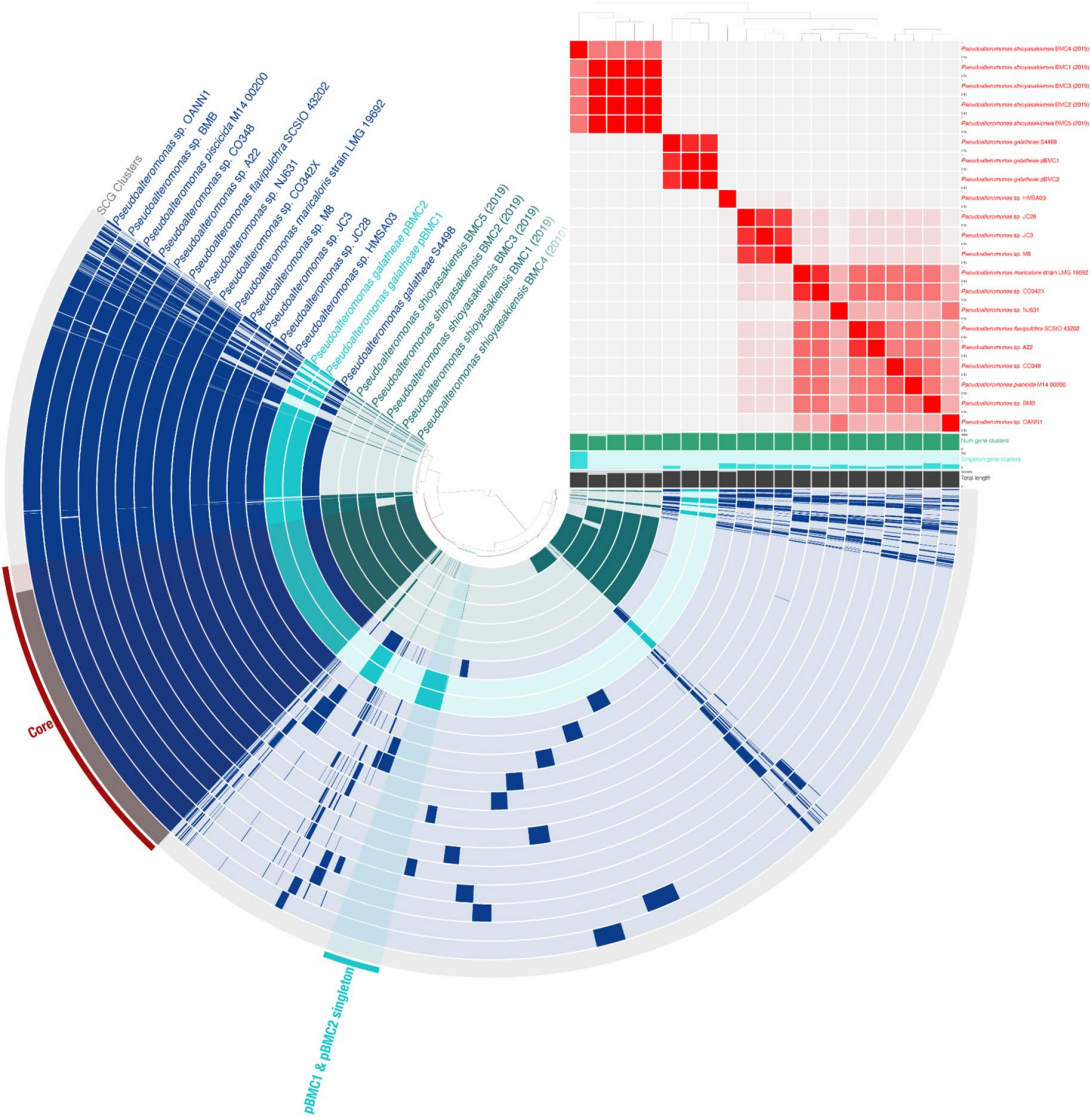
Pangenomic analysis of *Pseudoalteromonas galatheae* pBMC1 and pBMC2, other *Pseudoalteromonas* sp. strains from their phylogenomic clade in Fig. 1, and the *Pseudoalteromonas* sp. BMC1–5 strains from Rosado and colleagues^26,172^. The outermost circle displays single-copy genes (SCG cluster) shared by all genomes. The presence and absence of coding sequences in the genomes are represented in dark blue and faded dark blue, respectively, for each strain. The average nucleotide identity (95%–100%) comparison between the 21 genomes is shown in the heat map on the upper right. The dendrogram above the heat map shows the hierarchical clustering of genomes based on the occurrence of gene clusters (dark blue, below the heat map), number of singleton gene clusters (turquoise blue, below the heat map), and total length (gray, below the heat map) of each genome.

**Figure 7:**
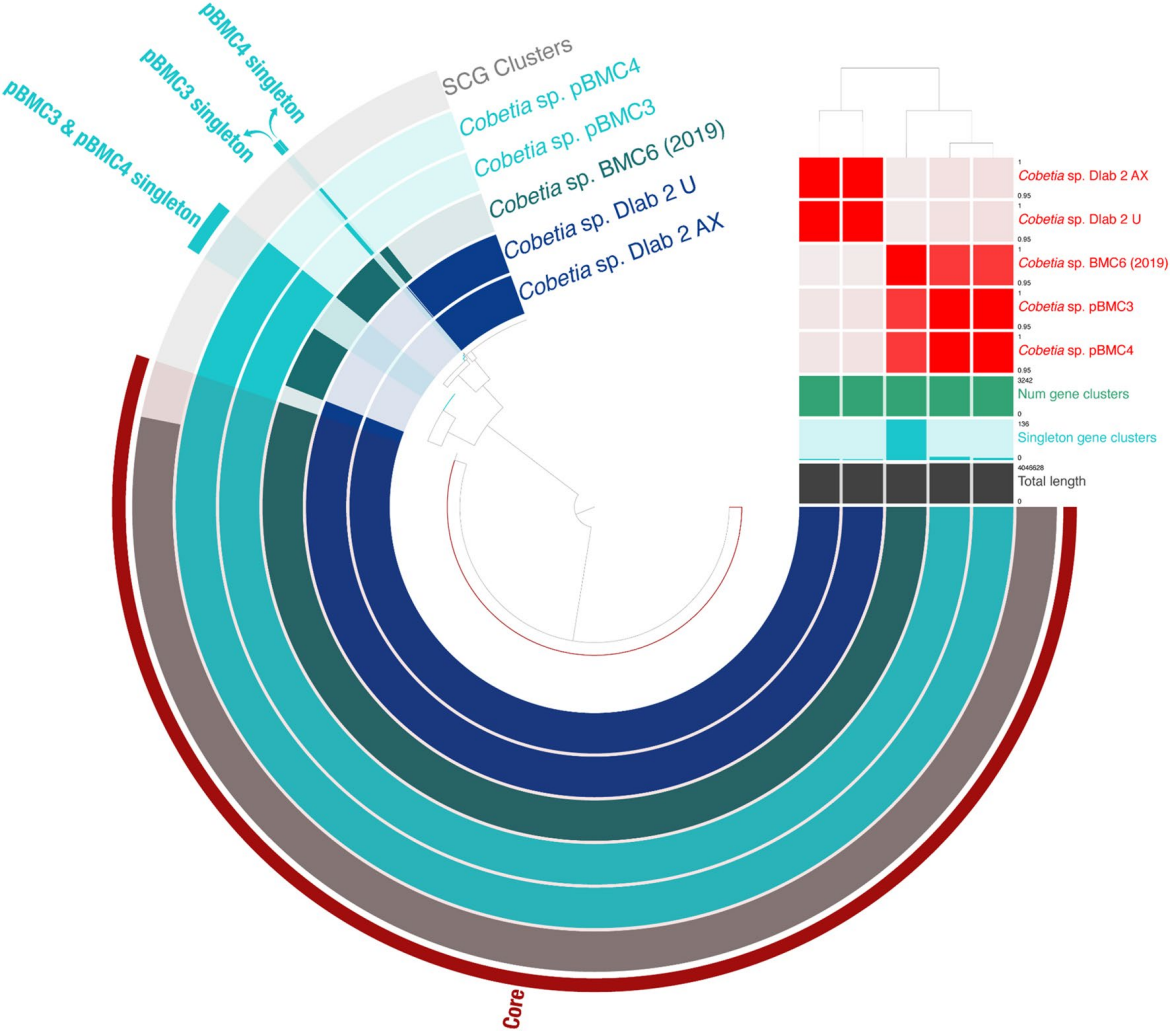
Pangenomic analysis of *Cobetia* sp. pBMC3 and pBMC4, the closest *Cobetia* sp. strains from the phylogenomic tree shown in Fig. 2, and the *Cobetia* sp. BMC6 strain from Rosado and colleagues^172^. The outermost circle displays single-copy genes (SCG cluster) shared by all genomes. The presence and absence of coding sequences in the genomes are represented in dark blue and faded dark blue, respectively, for each strain. The average nucleotide identity (95%–100%) comparison between the five genomes is shown in the heat map on the upper right. The dendrogram above the heat map shows the hierarchical clustering of genomes based on the occurrence of gene clusters (dark blue, below the heat map), number of singleton gene clusters (turquoise blue, below the heat map), and total length (gray, below the heat map) of each genome.

**Figure 8:**
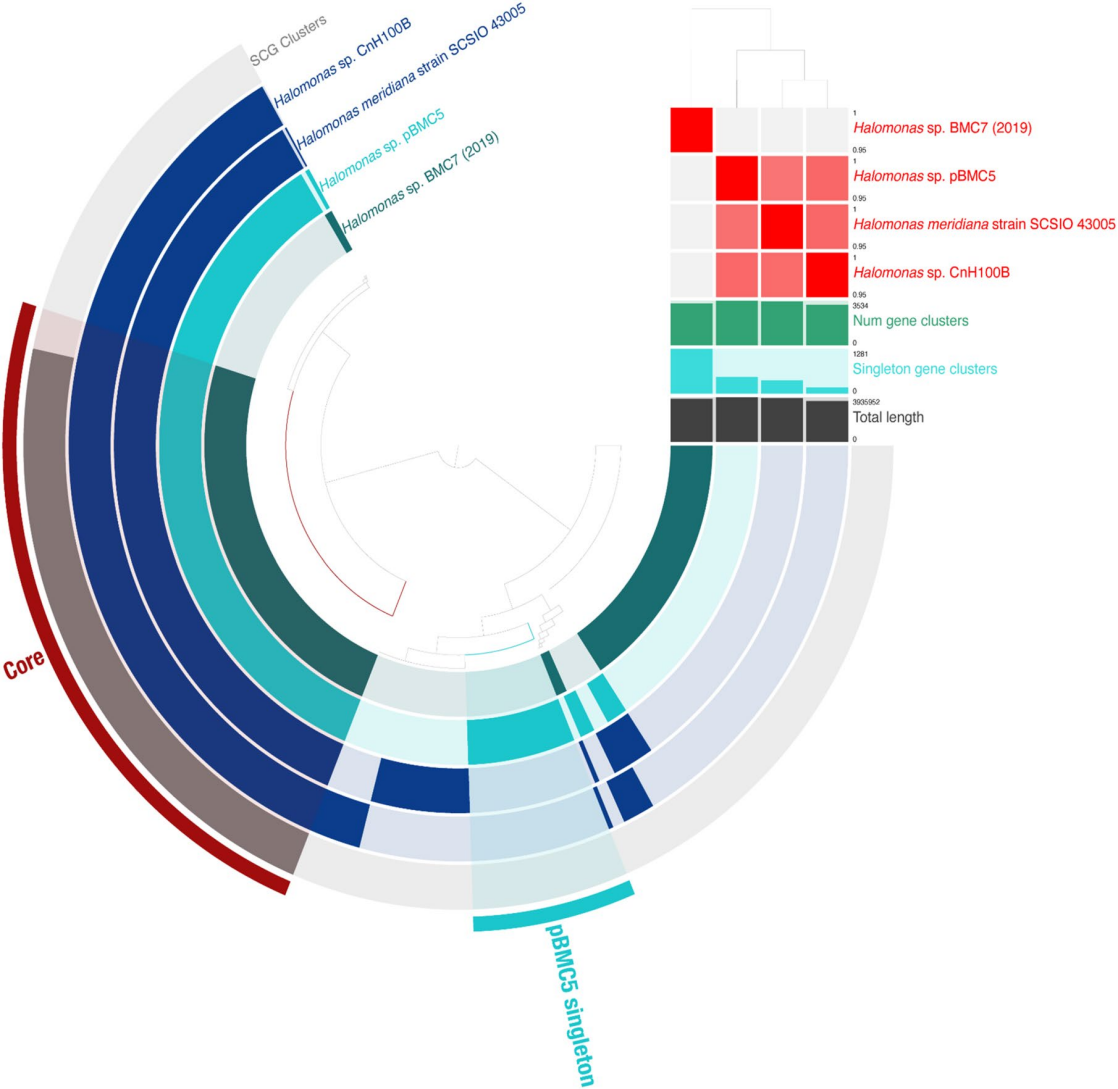
Pangenomic analysis of *Halomonas sp.* pBMC5 and other *Halomonas* sp. strains from its phylogenomic clade in Fig. 3 and *Halomonas* sp. BMC7 from the study by Rosado and colleagues^172^. The outermost circle displays single-copy genes (SCG cluster) shared by all genomes. The presence and absence of coding sequences in the genomes are represented in dark blue and faded dark blue, respectively, for each strain. The average nucleotide identity (95%–100%) comparison between the four genomes is shown in the heat map on the upper right. The dendrogram above the heat map shows the hierarchical clustering of genomes based on the occurrence of gene clusters (dark blue, below the heat map), number of singleton gene clusters (turquoise blue, below the heat map), and total length (gray, below the heat map) of each genome.

**Figure 9:**
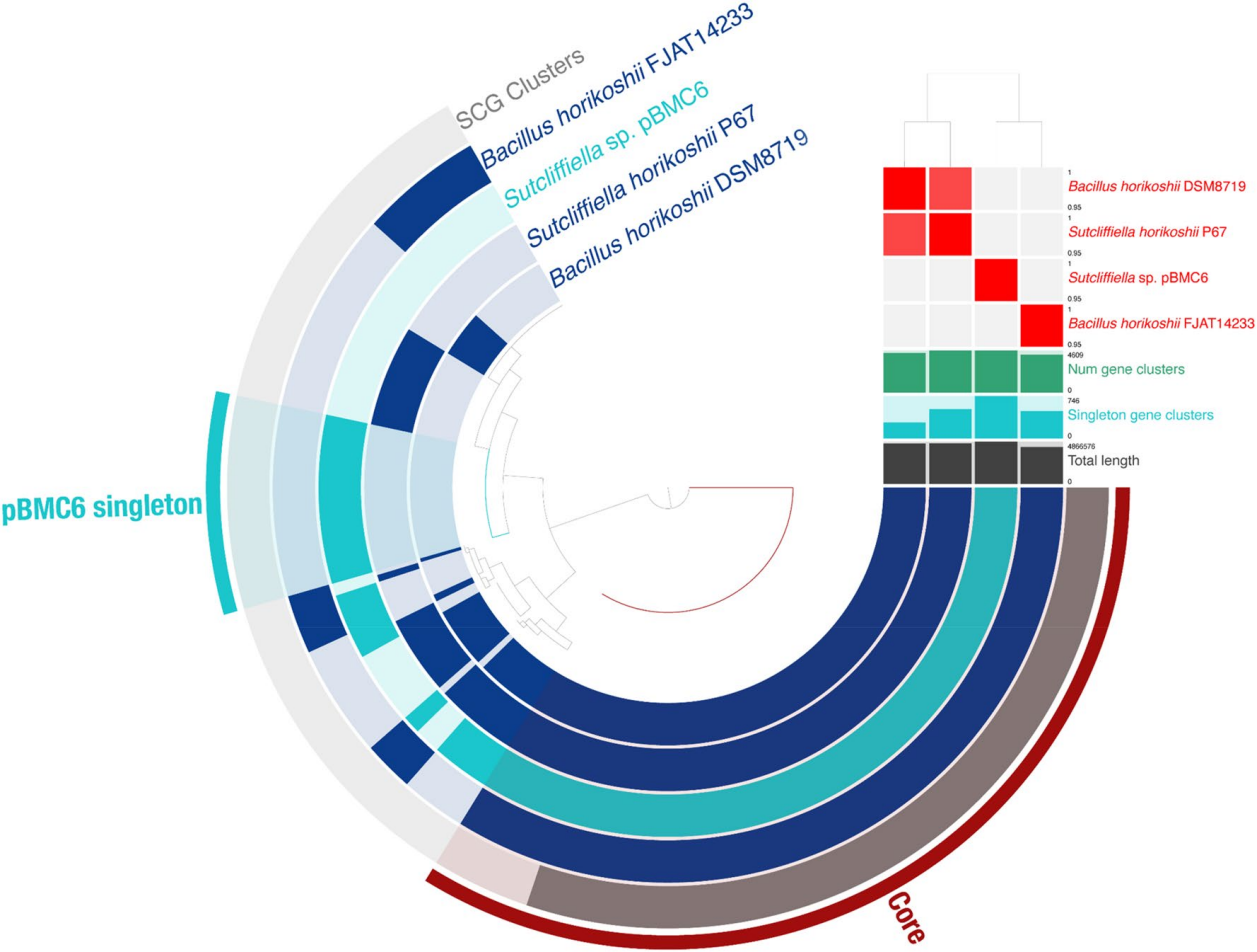
Pangenomic analysis of *Sutcliffiella* sp. pBMC6 and other *Sutcliffiella* sp. and *Bacillus* sp. strains from its phylogenomic clade in Fig. 4. The outermost circle displays single-copy genes (SCG cluster) shared by all genomes. The presence and absence of coding sequences in the genomes are represented in dark blue and faded dark blue, respectively, for each strain. The average nucleotide identity (95–100%) comparison between the four genomes is displayed in the heat map on the upper right. The dendrogram above the heat map shows the hierarchical clustering of genomes based on the occurrence of gene clusters (dark blue, below the heat map), number of singleton gene clusters (turquoise blue, below the heat map), and total length (gray, below the heat map) of each genome.

A pangenomic analysis was conducted for each pBMC clade to screen for genes unique to pBMC strains (Figs. 6, 7, 8 and 9). For the pangenome of pBMC1 and pBMC2, 19 *Pseudoalteromonas* sp. strains were added, including BMC from the study by Rosado et al. (2018) (Fig. 6). We identified 714 unique genes (i.e., 714 genes were present in the genomes of pBMC1 or pBMC2 but absent in the genomes of other 19 Pseudoalteromonas sp. strains). Of these 714 unique genes (450 of which were shared between pBMC1 and pBMC2), 604 were classified as hypothetical proteins, and the remaining 110 had known functions. Of the 110 genes with known functions, 49 were shared by pBMC1 and pBMC2. Furthermore, 3 of these 110 unique pBMC genes encoded proteins related to previously described beneficial traits for corals (Table 1).

**Table 1:** Proteins likely to benefit corals that were only identified in *Pseudoalteromonas galatheae* pBMC1 and pBMC2, *Cobetia amphilecti* pBMC3 and pBMC4, *Halomonas* sp. pBMC5, and *Sutcliffiella* sp. pBMC6 pangenomes. Functions retrieved from UniProt.

Protein	Function	pBMC
3-dehydroshikimate dehydratase	Involved in the biosynthesis of petrobactin, a catecholate siderophore that functions in both iron acquisition and virulence (PubMed: 17,189,355, PubMed: 18,955,706)	6
4-hydroxy-2-oxovalerate aldolase	Involved in the meta-cleavage pathway for the degradation of aromatic compounds such as phenols, cresols, and catechols	6
4,4'-diapolycopen-4-al dehydrogenase	Carotenoid biosynthesis	6
4,4'-diapolycopene oxygenase	Involved in the biosynthesis of C30 carotenoids. It has moderate to low activity on the C40 substrates neurosporene and lycopene	6
4,4'-diaponeurosporen-aldehyde dehydrogenase	Involved in the biosynthesis of the yellow-orange carotenoid staphyloxanthin, which plays a role in virulence via its protective function against oxidative stress	6
Anaerobic nitric oxide reductase transcription regulator NorR	Nitrogen metabolism. Required for the expression of anaerobic nitric oxide (NO) reductase	6
Chemotaxis protein CheV	Involved in the transmission of sensory signals from the chemoreceptors to the flagellar motors. Chemotaxis involves both a phosphorylation-dependent excitation and a methylation-dependent adaptation. CheV and CheW are involved in coupling the methyl-accepting chemoreceptors to the central two-component kinase CheA; they are both necessary for efficient chemotaxis	6
Cobalamin biosynthesis protein CobD	Converts cobyric acid to cobinamide by adding aminopropanol to the F carboxylic group	6
Corrinoid adenosyltransferase MMAB	Converts cob(I)alamin to adenosylcobalamin (adenosylcob(III)alamin), a coenzyme for methylmalonyl-CoA mutase, therefore participates in the final step of the vitamin B12 conversion	6
Denitrification regulatory protein NirQ	Nitrogen Cycle. Activator of nitrite and nitric oxide reductases	6
Fe/S biogenesis protein NfuA	Involved in iron-sulfur cluster biogenesis. Binds a 4Fe-4S cluster, can transfer this cluster to apoproteins, thereby intervening in the maturation of Fe/S proteins. It could also act as a scaffold/chaperone for damaged Fe/S proteins	1
Flavohemoprotein	Is involved in NO detoxification in an aerobic process, termed nitric oxide dioxygenase (NOD) reaction that utilizes O2 and NAD(P)H to convert NO to nitrate, which protects the bacterium from various noxious nitrogen compounds. Therefore, plays a central role in the inducible response to nitrosative stress	6
Gamma-glutamylcyclotransferase family protein YtfP	May play a role in antibiotic biosynthesis	5
Glycerophosphodiester phosphodiesterase	Glycerophosphodiester phosphodiesterase catalyzes the degradation of diphenyl phosphate (DPHP) to phenyl phosphate (PHP) (PubMed: 33,758,739). DPHP is an aryl phosphate ester used as a chemical additive and an industrial catalyst that can easily spread to the environment and exhibits toxicity toward organisms (PubMed:33,758,739)	6
GTP 3',8-cyclase	Catalyzes the cyclization of GTP to (8S)-3', 8-cyclo-7, 8-dihydroguanosine 5'-triphosphate (By similarity). Required for both nitrate assimilation and respiration	6
Homoserine O-acetyltransferase	Involved in the biosynthesis of the antibiotic D-cycloserine (DCS), a cyclic structural analog of D-alanine, used as an antitubercular agent. Catalyzes the transfer of the acetyl group from acetyl-CoA to the hydroxyl group of L-serine to yield the activated serine, O-acetyl-L-serine	6
HTH-type transcriptional regulator CysL	Transcriptional activator of the cysJI operon which is involved in sulfur assimilation	5, 6
HTH-type transcriptional regulator SutR	Regulates the expression of 12–16 transcription units involved in various steps of sulfur utilization. Represses expression of pfkB, fliZ, cysE, ydcO and its own expression. Activates expression of ypfN. Acts by binding to SutR boxes	2
Low molecular weight protein-tyrosine-phosphatase YfkJ	Dephosphorylates the phosphotyrosine-containing proteins. Involved in ethanol stress resistance	6
Mercuric reductase	Resistance to Hg^2+^ in bacteria appears to be governed by a specialized system which includes mercuric reductase. MerA protein is responsible for volatilizing mercury as Hg_0_	5
Nitric oxide synthase oxygenase	Catalyzes the production of nitric oxide	6
Peptide methionine sulfoxide reductase MsrA	Could have an important function as a repair enzyme for proteins that have been inactivated by oxidation. Catalyzes the reversible oxidation–reduction of methionine sulfoxide in proteins to methionine	6
Phospholipase YtpA	Phospholipase involved in the biosynthesis of the antibiotic bacilysocin. It probably catalyzes the hydrolysis of the 2-sn-acyl moiety of phosphatidylglycerol to produce bacilysocin (lysophosphatidylglycerol)	6
Protein-glutamate methylesterase/protein-glutamine glutaminase	Involved in chemotaxis. Part of a chemotaxis signal transduction system that modulates chemotaxis in response to various stimuli. Catalyzes the demethylation of specific methylglutamate residues introduced into the chemoreceptors	1, 6
Probable quorum-quenching lactonase YtnP	Probable hydrolase that is able to inhibit the signaling pathway required for the streptomycin production and development of aerial mycelium in S.griseus. Thus, serves as a defensive strategy against competing bacteria	6
Response regulator MprA	Member of the two-component regulatory system MprB/MprA which contributes to maintaining a balance among systems involved in stress resistance and is required for the establishment and maintenance of persistent infection in the host. MprB/MprA is involved in the regulation of numerous stress-responsive genes, including up-regulation of two sigma factors, sigE and sigB as well as pepD and mprA, and repression of multiple genes from regulons associated with hypoxia, starvation, and iron metabolism. The majority of genes regulated by MprB/MprA under a particular stress condition are different from those induced during normal growth, but several genes are commonly regulated under more than one condition	6
Riboflavin synthase	Catalyzes the dismutation of two molecules of 6,7-dimethyl-8-ribityllumazine, resulting in the formation of riboflavin (Vitamin B2) and 5-amino-6-(D-ribitylamino)uracil	6
RNA polymerase sigma factor YlaC	Sigma factors are initiation factors that promote the attachment of RNA polymerase to specific initiation sites and are then released. This sigma factor contributes to oxidative stress resistance	6
RsbT co-antagonist protein RsbRC	One of 4 functionally non-identical RsbR paralogs, it functions in the environmental signaling branch of the general stress response	6
Sensor histidine kinase LiaS	Member of the two-component regulatory system LiaS/LiaR probably involved in response to a subset of cell wall-active antibiotics that interfere with the lipid II cycle in the cytoplasmic membrane (bacitracin, nisin, ramoplanin and vancomycin). Seems also involved in response to cationic antimicrobial peptides and secretion stress	6
Thiamine-monophosphate kinase	Catalyzes the ATP-dependent phosphorylation of thiamine-monophosphate (TMP) to form thiamine-pyrophosphate (TPP), the active form of vitamin B1	6
Toluene efflux pump periplasmic linker protein TtgG	The periplasmic linker component of an organic solvent efflux pump. Involved in the export of a number of organic solvents, including toluene and styrene. This is the most important solvent efflux pump in this strain, although it can export AMP and some antibiotics	5

There were 32 singleton genes identified in the pangenome of pBMC3 and pBMC4, which included 3 other *Cobetia* sp. strains (Fig. 7). Of these 32 singleton genes (with no genes being shared between pBMC3 and pBMC4), 5 had known functions, and the remaining were classified as hypothetical proteins. Of these 5 unique pBMC genes, none encoded proteins related to previously assigned beneficial traits for corals (Table 1).

*Halomonas* sp. pBMC5 formed a clade with two other *Halomonas* sp. strains, and a pangenome involving these three strains and *Halomonas* sp. BMC7 from the study by Rosado and colleagues^26^ was established (Fig. 8). A total of 740 genes were identified as singletons, which were present in the pBMC5 genome alone. Among these 740 genes, 536 were classified as hypothetical proteins, and 204 had known functions. Of these 204 unique pBMC5 genes, 4 encoded proteins related to previously assigned and potentially novel beneficial traits for corals related to antibiotic synthesis and transport and sulfur assimilation (Table 1).

*Sutcliffiella* sp. pBMC6 formed a clade with another *Sutcliffiella* sp. and one *Bacillus* sp. strain (Fig. 4). For a more comprehensive pangenome, *B. horikoshii* strain FJAT-14233 was added to the above-mentioned strains (Fig. 9). This pangenome harbored the highest number of unique pBMC genes, with 3088 genes present only in the pBMC6 genome. Of these, 1755 were classified as hypothetical proteins, and the remaining 1333 had known functions. Among these 1333 genes, 27 encoded proteins related to previously assigned and potentially new beneficial traits for corals related to siderophore, carotenoid, vitamin, and antibiotic synthesis, nitrogen, sulfur and iron metabolism, chemotaxis, degradation of toxic compounds and stress resistance (Table 1).

### Biosynthesis of natural product

Mining of the six pBMC genomes on antiSMASH to screen for secondary metabolite BGCs revealed 49 BGCs across the six pBMC genomes. These included terpene (*n* = 2), type I and III polyketide synthase (PKS; *n* = 9 and *n* = 1, respectively), ectoine (*n* = 3), nonribosomal peptide synthetases (NRPSs; *n* = 8), nonribosomal peptide metallophores (*n* = 4), opine-like zincophores (*n* = 1), NRPS-independent (NI) IucA/IucC-like siderophores (*n* = 3), beta-lactone (*n* = 1), aryl polyene (*n* = 2), class I and IV lanthipeptides (both, *n* = 2), and lasso peptide (*n* = 1) as well as some putative clusters.

The NI siderophore cluster found in pBMC3 and pBMC4 showed 75% homology with known BGCs encoding desferrioxamine E (BGC0001572, BGC0001478, NCBI: MH015039.1). The ectoine cluster from pBMC5 showed 75% homology with several known ectoine-coding BGCs (BGC0000855, BGC0000856, BGC0000859, NCBI: DQ238213.1). The ectoine cluster of pBMC3 and pBMC4 showed 66% homology with the ectoine BGC000852 (NCBI: AF316874.1). The opine-like metallophore BGC found in pBMC6 showed 100% similarity with a known BGC encoding bacillopaline (BGC0002488, NCBI: CP002869.1), and the lasso peptide cluster showed 80% homology with the known BGC encoding paeninodin (BGC0001356, NCBI: AHKH01000064.1). Two terpenoid clusters were found in pBMC6. The first showed low homology with known clusters, highlighting the importance of continuing to study the biosynthesis of this potentially novel natural product in marine organisms. The other terpenoid gene cluster showed higher (66%) homology with a known BGC encoding a carotenoid (BGC0000645, NCBI: FJ040212.1). The pBMC6 genome contained a type III PKS cluster, and all other strains contained a type I-PKS cluster.

A protein family (Pfam)-based analysis was performed using antiSMASH, revealing squalene and phytoene synthase in *Sutcliffiella* sp. pBMC6 and a carotenoid biosynthesis-encoding gene (Table S4). The genomes of *P. galatheae* pBMC1 and pBMC2 contained lanthionine synthetase and lantibiotic biosynthesis-encoding genes as well as thioesterase synthase, which is necessary for peptide antibiotic production (Table S4). *Cobetia* sp. pBMC3 and pBMC4 harbored Pfam domains related to siderophore production and vitamin B12 and ectoine biosynthesis (Table S4), and ectoine synthase-encoding genes were present in *Halomonas* sp. pBMC5.

## Discussion

### Halomonas sp. pBMC5 and Sutcliffiella sp. pBMC6 as candidate novel species

Our results, particularly those based on the ANI and DDH values, suggest that both pBMC5 and pBMC6 are novel species, and further characterization is planned. The systematic classification of the family Bacillaceae has undergone numerous modifications in recent years due to the implementation of new taxonomic polyphasic techniques^80,81^. This has resulted in the creation of new genera from the previously classified *Bacillus* genus, such as the new genus *Sutcliffiella*^81^, and justifies the presence of *B. horikoshii* strains in the phylogenomic tree of pBMC6—likely an older classification of the current *S. horikoshii*—and the dominance of *Bacillus* sp. genomes in this tree. This is further corroborated by the presence of two putative prophages in pBMC6 that were predicted to infect *S. horikoshii* hosts.

### Genome screening reveals previously proposed beneficial traits of pBMC

Following the classification of each pBMC for identification and phylogenomic analysis, we screened the pBMC genomes for genes encoding proteins that are potentially beneficial for corals. We screened for genes related to catalase, urease, and siderophore production; phosphate assimilation; and nitrogen cycle and DMSP degradation through biochemical tests and PCR assays^44^, which are typically employed for BMC selection. We also detected genes involved in other potential beneficial traits (Table S3), including those related to oxidative stress, such as superoxide dismutases (all pBMC genomes), which exert an antioxidant effect by catalyzing the dismutation of superoxide (an ROS molecule that causes cell damage)^84^; catalase KatE (all pBMC genomes) and catalase-peroxidase KatG (pBMC1, pBMC2, pBMC5, and pBMC6 genomes), both of which protect cells from the toxic effects of H_2_O_2_ and aerated growth conditions^85–89^; manganese catalase (pBMC5 and pBMC6 genomes), which is also involved in the protection of cells from H_2_O_2_^90,91^; and glutathione synthetase (all pBMC genomes except for pBMC6), which produces glutathione that can subsequently be used by glutathione peroxidase (all pBMC genomes except for pBMC3 and pBMC4) to scavenge ROS, such as H_2_O_2_^92,93^. When seawater temperatures rise, the coral holobiont produce ROS, resulting in cell damage in both host and its symbionts^94–96^. A direct correlation between bleaching and ROS production has been previously reported^3^, and ROS-scavenging pBMC strains were hypothesized to mitigate coral bleaching^33^, making this a crucial trait when selecting pBMC.

Several genes involved in vitamin B-complex biosynthesis were found in our pBMC genomes, such as riboflavin synthase (all pBMC), pyridoxine 5’-phosphate synthase (all pBMC except for pBMC6), biotin synthase (all pBMC), dihydrofolate synthase and thymidylate synthase (all pBMC), and cobalamin synthase (all pBMC except for pBMC3 and pBMC4) for the biosynthesis of vitamins B2, B6, B7, B9, and B12, respectively. Vitamin B2 is necessary for glutathione reductase activity, which is involved in stress reduction by increasing antioxidant potential, and B2 deficiency increases lipid peroxidation^97^. Vitamin B6 catalyzes approximately 2% of all prokaryotic functions^98^, but it has not been widely studied in the marine environment^99,100^. It acts as an antioxidant during light exposure and against oxidative stress^101,102^. Vitamin B7 is a cofactor in several metabolic pathways, such as fatty acid biosynthesis, amino acid metabolism, and gluconeogenesis^103^. Vitamin B12 is involved in several metabolic pathways, including the production of the antioxidants glutathione and DMSP^104^, which are important for neutralizing high concentrations of ROS generated from heat stress events^33,43^. Bacteria that exist in association with corals possess genes encoding for proteins related to the biosynthesis of essential vitamins, such as B1, B2, and B7, whereas their coral host does not have the capacity to produce them^105^. This suggests that the coral holobiont can only take up these essential vitamins through heterotrophic feeding and/or from its bacterial symbionts. Furthermore, coral symbionts from the family Symbiodiniaceae are auxotrophs for various B-complex vitamins, which they acquire from exogenous sources such as bacteria^83,104, 106–108^. This highlights the important role of bacterial symbionts in ensuring coral health. For these reasons and because of the close interaction between several B-complex vitamins, the presence of genes encoding proteins related to B-complex vitamin biosynthesis is suggested as a BMC trait.

We also screened for other genes related to metabolism. Siderophore synthase was present in pBMC6. This enzyme produces siderophores that can scavenge iron from the environment, a trait that is beneficial for corals^33,82^ and other organisms^109^. In general, the bioavailability of iron in oceans is extremely low; consequently, the growth and survival of organisms that use iron for essential physiological processes, such as photosynthesis and nitrogen fixation, cannot be guaranteed^110,111^. Thus, bacteria that exist in association with other marine organisms, such as corals and microalgae, produce siderophores to capture and concentrate iron into a bioavailable form that can be used by other organisms^112,113^ Apart from siderosphere production, we also found that some of our pBMC produced ectoine (pBMC3, pBMC4, and pBMC5) and betaine (pBMC1, pBMC2, pBMC3, pBMC4, and pBMC5), which have been previously described in BMC genomes and proposed as compounds that plays a role in beneficial mechanisms in corals^82^. Ectoines and betaines are important for osmoregulation and act as protective agents under thermal stress and high irradiance^114–116^. They also contribute to the nitrogen biomass of corals in reefs^117^. In marine microalgae, the ectoine content was found to increase in the presence of bacteria, highlighting the crucial role of these microorganisms in host health^118^. Betaine and ectoine production significantly improves environmental stress tolerance, including pH stress^119^ and heat stress^120^ in aquatic organisms^121^, such as corals and their symbionts^116^. Ectoines can help mitigate the harmful effects of heat stress, high salinity, ROS, and radiation^122^. Pei et al. identified betaine lipids as leading metabolite drivers for differentiating heat-bleached corals from healthy ones, revealing new tools to screen for heat-resistant corals and their symbionts, such as BMC^116,123^.

We also found genes involved in the nitrogen cycle, including nitrate reductase (pBMC3, pBMC4, and pBMC5) and cyanate hydratase (pBMC5). The presence of these genes was previously proposed as a BMC trait^33,43^ because balancing this nutrient’s availability contributes to maintaining desirable levels of bioavailable nitrogen, limiting algal growth and leading to an accumulation of photosynthates in algal cells that, when released, feed the coral host and promote its growth. Additionally, increased coral catabolic activity due to an environmental stressor leads to host starvation and increased nitrogen availability to Symbiodiniaceae members of the holobiont, potentially causing destabilization of the host’s nutrient cycle and of the Symbiodiniaceae–coral interaction^10,124^.

Screening for genes related to DMSP degradation and sulfur metabolism revealed the presence of DMSP CoA transferase/lyase DddD (pBMC3 and pBMC4) and acryloyl-CoA reductase AcuI/YhdH (BMC1 and pBMC2). DMSP is found in several marine organisms, including Symbiodiniaceae^107,125^, and is a ROS scavenger in marine algae^126^, an attribute that has been proposed as a BMC trait due to its antioxidant activity^33^. Mechanisms of DMSP breakdown have also been hypothesized as BMC traits because a high DMSP concentration can lead to dysbiosis and signal the location of more vulnerable coral to pathogens through chemotaxis^18,33, 127^.

### Discovery of the presence of BMC-associated prophages

Prophages are DNA from bacteriophages (or bacteria-infecting viruses) that are integrated into the genomes of their bacterial host upon infection. They are mainly studied as contributors to the virulence of pathogenic reef bacteria, such as *V. coralliilyticus*, because they encode virulence factors^128^. The detection of putative prophages in two of our six pBMC strains revealed the potentially presence of prophages across marine bacteria taxa, and also implies a positive role of prophages associated with beneficial bacteria. As prophages also protect the bacterial host against virulent phage infections via superinfection exclusion^129^, pBMC containing prophages may have a competitive advantage against other bacteria, including pathogens. Thus, prophages might both expand the metabolic capabilities and protection of beneficial bacteria, and further research is warranted.

### Pangenomes exhibit novel pBMC beneficial traits and other applicable functions

We conducted pangenomic analyses that included our pBMC strains and strains closely related to them in the phylogenomic trees to screen for genes unique to our pBMC strains. In the *Pseudoalteromonas* pangenome, 110 genes with known functions were only present in the pBMC genomes (i.e., they were present in one or both of our *P. galatheae* pBMC strains). This included a protein involved in chemotaxis and some proteins involved in iron/sulfur metabolism. Chemotaxis may be an important BMC trait because it was previously suggested to play a crucial role in defining patterns of microbial diversity, coral metabolism, coral infection dynamics, and chemical cycling processes, thereby influencing coral holobiont health^130^. Despite the absence of DMSP-related genes in the genomes of the *Pseudoalteromonas* sp. pBMC strains compared with the other *Pseudoalteromonas* sp. strains, the metabolism and production of sulfur compounds has been proposed as a BMC trait because they inhibit the growth of coral pathogens and also play a role in the structure of bacterial communities of the coral holobiont^33^.

In the *Cobetia* pangenome, we found five genes with known functions that were only present in the pBMC genomes, but none of them showed a clear benefit to the host. In the *Halomonas* pangenome, we found 204 genes with known functions that were only present in the pBMC5 genome, inclunding genes involved in carotenoid biosynthesis, antibiotic biosynthesis, and sulfur metabolism. Although not directly related to host health but more to the environment as a whole, the presence of mercury resistance-related genes in the pBMC5 genome is of crucial interest. Heavy metals are known to be environmental toxins due to their bioaccumulation in the food chain, becoming increasingly hazardous for the higher trophic levels^131,132^ The use of bioremediation for the removal of toxic metals has been studied, but only a few studies were performed in a marine environment^133^. The trend is similar for mercury-resistant marine bacteria, and the use of mercury-resistant marine bacteria for bioremediation of mercury contamination has received little attention^134^. However, their use is associated with certain advantages, such as simple process, lower amount of secondary metabolites, and lower cost than commonly used chemical technologies as well as better adaptability and higher resistance to adverse environmental conditions compared with terrestrial bacteria^134,135^. Some studies have reported that mercury-resistant marine bacteria show a higher capability for mercury bioremediation and can reduce the toxic effects of mercury in contaminated environments^134,136^.

We found that the *Sutcliffiella* genome had the highest amount of singleton genes of all the pangenomes generated in this study, with 1333 genes with known functions present only in the pBMC6 genome, including genes encoding proteins related to the biosynthesis of siderophores, carotenoids, antibiotics, vitamins B1, B2 and B12; nitrogen, iron, and sulfur metabolism; chemotaxis; and oxidative stress resistance. Other than the abovementioned genes, we identified a gene that was involved in the detoxification of reactive aldehydes, which are highly reactive organic chemical compounds that mostly arise due to oxidative stress^137^. We also identified proteins involved in the degradation of aromatic compounds, such as phenols, cresols, catechols, and diphenyl phosphate (DPHP). Phenols and cresols are harmful to the environment^138^, and catechol bioaccumulation negatively affects the entire ecosystem^139^. DPHP is used as a chemical additive in numerous industrial products, and because it does not bind with other chemicals, it is easily spread to the environment, where it has been widely detected^140–142^. When in the environment, DPHP has a long half-life and is immunotoxic and neurotoxic to other organisms^143^. Despite not being comprehensively studied in marine organisms, recent studies have reported its negative effects on fish growth, energy metabolism, and reproduction^144–147^. Further investigations are necessary to understand the toxic and negative effects of DPHP on corals, and it may be beneficial to possess the necessary genetic machinery to degrade such harmful compounds. Lastly, we identified a regulatory system that functions under specific stress conditions, such as hypoxia and starvation, and appears to be beneficial to host health and resilience.

### pBMC and the importance of their secondary metabolites

Secondary metabolites are important for defense against microorganisms, toxic compounds, and UV radiation as well as essential for symbiotic relationships^148^; they are abundantly found within the coral holobiont^149^. Accordingly, we assessed the BGCs and Pfams of our six pBMC strains using the antiSMASH platform, revealing 11 secondary metabolite production clusters: ectoine, beta-lactone, terpene, aryl polyene, lasso peptide, NRPS, nonribosomal peptide metallophores, NI siderophores, opine-like zincophores, class I and IV lanthipeptides, and type I and III PKS. Ectoines are important because they reduce the effects of heat stress, high salinity, ROS, and radiation. Beta-lactone derivatives are extremely diverse, comprising 30 distinct families, many of which have antimicrobial activity^150^, while others are important elements for antibiotic production^148^. Terpenes are diverse organic compounds that play a role in defense mechanisms in plants and fungi^151,152^, serving as antioxidants and protecting cells from oxidative stress^153^, and their presence indicates production of diverse bioactive compounds^154^. Some terpenes and carotenoids, such as squalenes, are pigments involved in photosynthesis and signaling. However, they also possess antioxidant activity and can neutralize oxidative stress^155–157^. Aryl polyenes are pigments that are structurally and functionally related to carotenoids and confer protection against photo-oxidative damage and lipid peroxidation^158,159^. Lasso peptides have antimicrobial and antiviral properties and also show thermal and chemical resistance^148,160^. NRPSs are sources of newly discovered antibacterial agents that have also been widely studied for their antiviral and anti-inflammatory properties^148,161^. Microorganisms scavenge metal ions from the environment via metallophores^162^. Some of these metallophores, such as siderophores like desferrioxamine E, play important roles in biocontrol and bioremediation^163–165^, and are considered beneficial when selecting for BMC^33^. Zinc is also an essential nutrient for several cellular processes and is taken from the surrounding environment by bacteria^166^. Similar to iron availability, zinc is also present at low concentrations in the environment and is captured by zincophores (also called opine-like zincophores in bacteria), such as bacillopaline, produced by microorganisms^167^. Lanthipeptides that possess antimicrobial activity are known as lantibiotics^168^, but their functions are not limited to this; they may also possess antifungal and antiviral properties^169,170^. The presence of at least one PKS BGC in each pBMC genome suggests that these bacterial strains have the necessary genomic tools to synthesize various polyketides that are likely to have beneficial properties^161^.

### Red Sea pBMC and their Indo-Pacific Ocean counterparts

Rosado and colleagues^26^ successfully manipulated the coral microbiome of *P. damicornis* by adding BMC to coral fragments in a mesocosm setting, and the genomes of these BMC were screened to identify potential beneficial mechanisms and/or traits based on previous literature^82^. Some of the BMC from the study by Rosado and colleagues^26,82^ share several gene functions related to putative beneficial traits for corals with some of our pBMC (e.g., superoxide dismutase, glutathione synthetase, catalase-peroxidase, adenosylcobinamide-phosphate synthase, adenosylcobinamide kinase, betaine aldehyde dehydrogenase, choline dehydrogenase, ectoine hydroxylase, L-ectoine synthase, nitrite reductase, and CoA transferase) (Table S3)^82^. However, when examining the pangenomes of each genus, we noted that our pBMC had a unique set of genes that were absent in the genomes of the BMC from the study by Rosado and colleagues^82^. Moreover, some of these genes represent potential beneficial traits or mechanisms (Figs. 6, 7, 8 and Table 1).

Our results revealed potential insights into how bacteria help corals during periods of stress. Although some of the abovementioned beneficial traits are hypothetical, others have already been validated in previous studies on the differential expression of genes during heat stress experiments in corals^18^. Moreover, the identification and classification of these beneficial features depend on and is limited to existing databases. Additionally, relying on cultivation-dependent methods to obtain candidate pBMC introduces an element of chance in discovering and identifying potential candidates. This can be mitigated by obtaining a large number of isolates and meticulously screening and testing them for beneficial traits. Notably, all pBMC examined in this study, even those from the same genus, were distinct and demonstrated potential to contribute to the health and resilience of corals, indicating the need for continued efforts to isolate^149,171,172^, explore^82,173–175^ and test^176,177^novel pBMC. We also highlight the discovery of prophages associated with two of the BMCs and their potential role in providing competitive advantage to coral probiotics against other bacteria.

## Supplementary Information

Supplementary Information.

## Data Availability

Scripts that were used in the assembly and evaluation of the study isolate genomes are available at https://zenodo.org/records/10774634. The raw reads and assemblies from this study are publicly available in the European Nucleotide Archive (study accession number PRJEB62849; https://www.ebi.ac.uk/ena/browser/view/PRJEB62849), and the respective sample accession numbers are as follows: (i) *Pseudoalteomonas galatheae* pBMC1, SAMEA114261728; (ii) *Pseudoalteomonas galatheae* pBMC2, SAMEA114261729; (iii) Cobetia sp. pBMC3, SAMEA114261730; (iv) Cobetia sp. pBMC4, SAMEA114261731; (v) Halomonas sp. pBMC5, SAMEA114261732; (vi) Sutcliffiella sp. pBMC6, SAMEA114261733. The genome assemblies have also been made available at https://zenodo.org/records/11204102.
