# Publisher Correction: Unlocking the genomic potential of Red Sea coral probiotics

**DOI:** 10.1038/s41598-024-75673-x

**Published:** 2024-10-15

**Authors:** Inês Raimundo, Phillipe M. Rosado, Adam R. Barno, Chakkiath P. Antony, Raquel S. Peixoto

**Affiliations:** Biological and Environmental Science and Biological and Environmental Science and Engineering Division (BESE), King Abdullah University of Science and Technology, Biological and Environmental Science and Engineering Division, Thuwal, Saudi Arabia

Correction to: *Scientific Reports *10.1038/s41598-024-65152-8, published online 24 June 2024

In the original version of this Article, the in-text citations numbers are incorrect starting from sentence “Additionally, the six pBMC genomes were searched for putative prophage sequences using the PHASTEST and VirSorter2 online analysis tools^63,64^.” in section “*Structural and functional annotation of pBMC genomes”.*

The original Article has been corrected.

