# Supplementary material for: Unlocking the genomic potential of Red Sea coral probiotics
Source: Sci Rep. 2024 Jun 24;14:14514. doi: 10.1038/s41598-024-65152-8 (PMC11196684; doi:10.1038/s41598-024-65152-8)
Supplement: Supplementary file 1 — Supplementary Information. [file 41598_2024_65152_MOESM1_ESM.pdf]

SUPPLEMENTARY MATERIAL

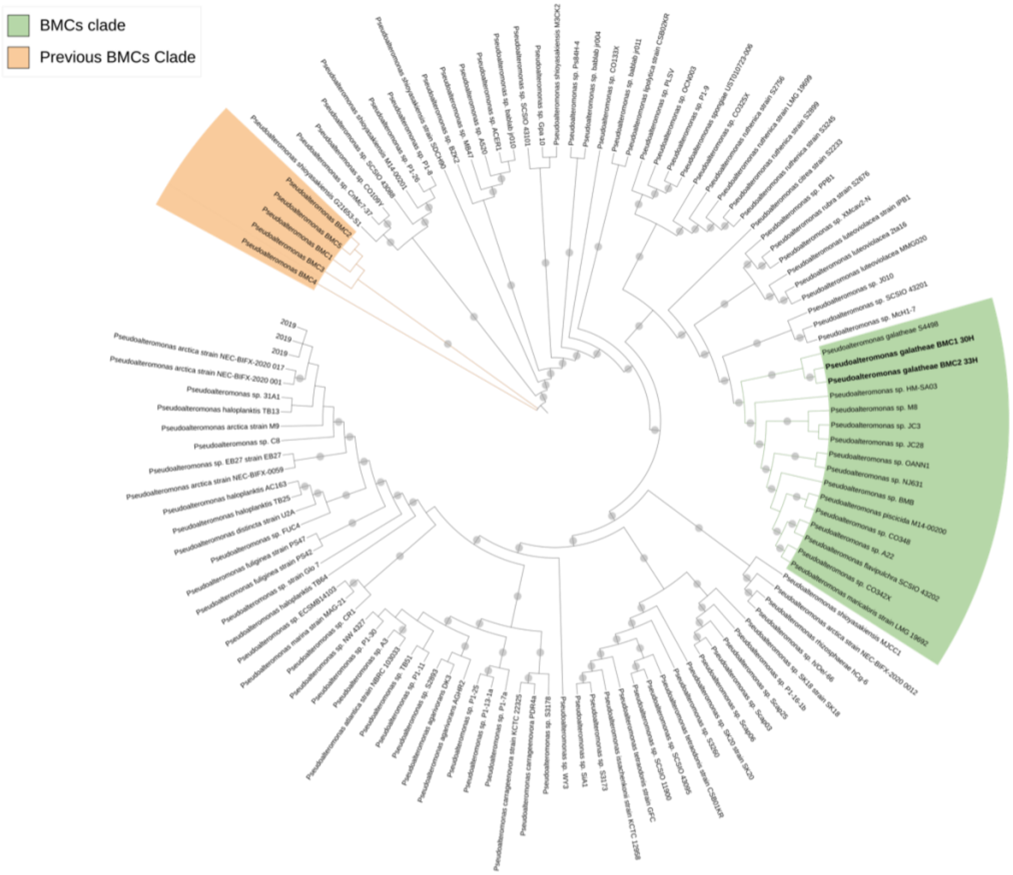

Figure S1. Phylogenomic inference of publicly available genomes from 113 *Pseudoalteromonas* sp. strains and the genomes of our two *Pseudoalteromonas galatheae* strains (pBMC1 and pBMC2; highlighted in bold), totaling 115 genomes. The tree was assembled by comparing 500 proteins using the codon tree method of the PATRIC platform. This method selects global protein families as homology groups and analyzes aligned proteins and DNA encoding single-copy genes using the RAxML program. Based on RAxML, the LG model was considered the best model to construct this tree. The green-shaded area represents the strains that form a monophyletic clade with pBMC1 and pBMC2. The orange-shaded area represents the monophyletic clade formed by the BMC strains from the study by Rosado *et al.* (2019). Gray dots on the branch length correspond to a bootstrap value of 100.

Table S1. Genome characteristics of our six putative beneficial microorganisms for corals (pBMC) strains.

Genome	Completeness (%)	Contamination (%)	Total number of reads	Number of contigs	G + C content (%)
Pseudoalteromonas_galatheae_pBMC1	99.89	1.22	264380	9	42.94
Pseudoalteromonas_galatheae_pBMC2	99.89	1.76	241222	6	42.98
Cobetia_sp._pBMC3	99.14	0.86	137126	2	62.32
Cobetia_sp._pBMC4	99.14	0.86	156058	2	62.31
Halomonas_sp._pBMC5	100.00	2.23	235549	2	57.94
Sutcliffiella_sp._pBMC6	97.99	1.54	345950	2	41.14

Table S2. Taxonomic classification of all genomes of our putative beneficial microorganisms for corals (pBMC) according to their phylogenomic trees using two different tools. The Genome-to-Genome Distance Calculator (GGDC) was used to calculate DNA–DNA hybridization (DDH) values, and FastANI was used to calculate the average nucleotide identity (ANI) values.

Genome 1	Genome 2	GGDC (DDH estimate %)	FastANI (OrthoANIu value %)
Pseudoalteromonas_galatheae_pBMC1	Pseudoalteromonas_galatheae S4498*	92.00	99.17
Pseudoalteromonas_galatheae_pBMC1	Pseudoalteromonas_galatheae_pBMC2	100.00	99.95
Pseudoalteromonas_galatheae_pBMC2	Pseudoalteromonas_galatheae S4498*	92.40	99.19
Pseudoalteromonas_galatheae_pBMC2	Pseudoalteromonas_galatheae_pBMC1	100.00	99.95
Cobetia_sp._pBMC3	Cobetia_sp._pBMC4	100.00	99.98
Cobetia_sp._pBMC3	Cobetia BMC6	94.80	98.95
Cobetia_sp._pBMC3	Cobetia sp. Dlab-2-AX	87.30	95.4
Cobetia_sp._pBMC3	Cobetia sp. Dlab-2-U	84.10	95.51
Cobetia_sp._pBMC4	Cobetia_sp._pBMC3	100.00	99.98
Cobetia_sp._pBMC4	Cobetia BMC6	94.90	98.84
Cobetia_sp._pBMC4	Cobetia sp. Dlab-2-AX	87.40	95.45
Cobetia_sp._pBMC4	Cobetia sp. Dlab-2-U	84.20	95.45
Halomonas_sp._pBMC5	Halomonas sp. CnH100-B	80.40	97.94
Halomonas_sp._pBMC5	Halomonas meridiana strain SCSIO 43005	76.80	97.94

Halomonas_sp._pBMC5	Halomonas meridiana strain R1t3	57.60	87.8
Halomonas_sp._pBMC5	Halomonas sp. 707B3	59.20	87.26
Halomonas_sp._pBMC5	Halomonas sp. R1t4	53.50	87.57
Halomonas_sp._pBMC5	Halomonas sp. R1t8	53.60	87.64
Halomonas_sp._pBMC5	Halomonas sp. Ps84H-12	52.70	87.56
Halomonas_sp._pBMC5	Halomonas meridiana DSM5425*	52.60	87.76
Sutcliffiella_sp._pBMC6	Sutcliffiella_horikoshii_P67	46.50	84.95
Sutcliffiella_sp._pBMC6	Bacillus_horikoshii_DSM_8719	47.60	84.87
Sutcliffiella_sp._pBMC6	Bacillus_horikoshii_strain_SJAT-14233	53.90	85.96

*Type strains.

Table S3. Examples of gene functions related to putative beneficial traits for coral found in the genomes of our putative beneficial microorganisms for corals (pBMC).

Products	pBMC1	pBMC2	pBMC 3	pBMC 4	pBMC 5	pBMC 6	Subsystem	Function
Catalase KatE (EC 1.11.1.6)	**Y**	**Y**	**Y**	**Y**	**Y**	**Y**	Oxidative stress	Protection from ROS
Catalase-peroxidase KatG (EC 1.11.1.21)	**Y**	**Y**	-	-	**Y**	**Y**	Oxidative stress	Bifunctional enzyme with catalase and peroxidase activity
Glutathione synthetase (EC 6.3.2.3)	**Y**	**Y**	**Y**	**Y**	**Y**	-	Oxidative stress	Synthesizes glutathione from L-cysteine and L-glutamate
Manganese catalase (EC 1.11.1.6)	-	-	-	-	**Y**	**Y**	Oxidative stress	Protection from ROS
Superoxide dismutase [Cu-Zn] precursor (EC 1.15.1.1)	-	-	**Y**	**Y**	**Y**	**Y**	Oxidative stress	Protection from ROS
Superoxide dismutase [Fe] (EC 1.15.1.1)	**Y**	**Y**	**Y**	**Y**	-	**Y**	Oxidative stress	Inactivates superoxide anion radicals that are normally produced within cells
Superoxide dismutase	**Y**	**Y**	-	-	**Y**	**Y**	Oxidative stress	Protection from ROS

[Mn] (EC 1.15.1.1)								
Siderophore synthetase	-	-	-	-	-	**Y**	Metabolism	Iron acquisition and metabolism (Anthrachelin biosynthesis protein AsbA; Siderophore synthetase superfamily, group A and large component, acetyltransferase)
Biotin synthase (EC 2.8.1.6)	**Y**	**Y**	**Y**	**Y**	**Y**	**Y**	Metabolism	Production of biotin (B7)
Cobalamin synthase (EC 2.7.8.26)	**Y**	**Y**	-	-	**Y**	**Y**	Metabolism	Cobalamin (Vitamin B12) synthesis. A cofactor involved in the production of the aminoacid methionine, needed to synthesize every protein as well as in diverse metabolic pathways (e.g., generation of antioxidants glutathione and DMSP)
Dihydrofolate synthase (EC 6.3.2.12)	**Y**	**Y**	**Y**	**Y**	**Y**	**Y**	Metabolism	Folate (vitamin B9) biosynthesis
Pyridoxine 5'-phosphate	**Y**	**Y**	**Y**	**Y**	**Y**	-	Metabolism	Pyridoxin (Vitamin B6) Biosynthesis

synthase (EC 2.6.99.2)								
Riboflavin synthase eubacterial/ eukaryotic (EC 2.5.1.9)	**Y**	**Y**	**Y**	**Y**	**Y**	**Y**	Metabolism	Riboflavin (vitamin B2), FMN, and FAD metabolism with fusion events
Thymidylate synthase (EC 2.1.1.45)	**Y**	**Y**	**Y**	**Y**	**Y**	**Y**	Metabolism	Folate (vitamin B9) biosynthesis
Nitrite reductase [NAD(P)H] large subunit (EC 1.7.1.4)	-	-	**Y**	**Y**	**Y**	-	Nitrogen cycle	Nitrite reduction
Nitrite reductase [NAD(P)H] small subunit (EC 1.7.1.4)	-	-	-	-	**Y**	-	Nitrogen cycle	Nitrite reduction
Nitrite reductase (NAD(P)H) large subunit, NirD	-	-	**Y**	**Y**	-	-	Nitrogen cycle	Nitrite reduction
Cyanate hydratase	-	-	-	-	**Y**	-	Nitrogen Metabolism	

(EC 4.2.1.104)								
L-ectoine synthase (EC 4.2.1.108)	-	-	**Y**	**Y**	**Y**	-	Ectoines synthesis	Part of ectoine biosynthesis
Acryloyl-CoA reductase AcuI/YhdH (EC 1.3.1.84)	**Y**	**Y**	-	-	-	-	Sulfur Metabolism	Bacterial pathways for DMSP and acrylate catabolism
Dimethylsulfoniopropionate CoA transferase/ lyase DddD, 3-hydroxypropionate generating	-	-	**Y**	**Y**	-	-	DMSP degradation	Dimethylsulfide (DMS) producer from DMSP degradation
Adenosylcobinamide kinase (EC 2.7.1.156) / Adenosylcobinamide-phosphate guanylyltransferase (EC 2.7.7.62)	**Y**	**Y**	-	-	**Y**	**Y**	Cobalamin synthesis	Involved in adenosylcobalamin biosynthesis, which is part of cofactor biosynthesis

Adenosylcobinamide-phosphate synthase (EC 6.3.1.10)	-	-	-	-	**Y**	**Y**	Cobalamin synthesis	Part of the cobalamin biosynthetic pathway
Betaine aldehyde dehydrogenase (EC 1.2.1.8)	**Y**	**Y**	**Y**	**Y**	**Y**	-	Betaine glycine synthesis	Part of the glycine betaine biosynthetic pathway
Choline dehydrogenase (EC 1.1.99.1)	**Y**	**Y**	**Y**	**Y**	**Y**	-	Betaine glycine synthesis	Part of the betaine biosynthesis pathway from choline
Ectoine hydroxylase	-	-	**Y**	**Y**	**Y**	-	Ectoines synthesis	Involved in the 5-hydroxyectoine biosynthesis
High-affinity choline uptake protein BetT	-	-	**Y**	**Y**	**Y**	-	Stress response, defense	Choline uptake and conversion to betaine clusters
Gluthatione peroxidase (EC 1.11.1.9)	**Y**	**Y**	**-**	**-**	**Y**	**Y**	Stress response, defense	Reduces hydrogen peroxide to water to limit its harmful effects.

DMSP, dimethylsulfoniopropionate; ROS, reactive oxygen species.

Table S4. Protein families (Pfams) that may be beneficial for the host and are present in the genomes of the six putative beneficial microorganisms for corals (pBMC) strains.

Accession	Name	Description	pBMC
PF05147	LANC-like	Lanthionine synthetase C-like protein	**1, 2**
PF14028	Lant_dehydr_C	Lantibiotic biosynthesis dehydratase C-term	**1, 2**
PF00975	Thioesterase	Thioesterase is required for the addition of the last amino acid to peptide antibiotics	**1, 2**
PF04183	IucA_IucC	IucA/IucC family catalyse steps in biosynthesis of the siderophore aerobactin	**3, 4, 6**
PF07683	CobW_C	Cobalamin synthesis protein cobW, C-terminal domain	**3, 4**
PF06339	Ectoine_synth	Ectoine synthase	**3, 4, 5**
PF04240	Caroten_synth	Carotenoid biosynthesis protein	**6**
PF00494	SQS_PSY	Squalene/phytoene synthetase	**6**
PF13243	SQHop_cyclase_C	Squalene-hopene cyclase C-terminal domain	**6**
PF13249	SQHop_cyclase_N	Squalene-hopene cyclase N-terminal domain	**6**
PF02797	Chal_sti_synt_C	Chalcone and stilbene synthases, C-terminal	**6**
PF00195	Chal_sti_synt_N	Chalcone and stilbene synthases, N-terminal	**6**
PF13690	CheX	Chemotaxis phosphatase CheX	**6**
PF10100	Staph_opine_DH	Staphylopine dehydrogenase	**6**
